# Carotenoid-based coloration predicts both longevity and lifetime fecundity in male birds, but testosterone disrupts signal reliability

**DOI:** 10.1371/journal.pone.0221436

**Published:** 2019-08-23

**Authors:** Alejandro Cantarero, Lorenzo Pérez-Rodríguez, Ana Ángela Romero-Haro, Olivier Chastel, Carlos Alonso-Alvarez

**Affiliations:** 1 Section of Ecology, University of Turku, Turku, Finland; 2 Instituto de Investigación en Recursos Cinegéticos, IREC (CSIC - UCLM - JCCM), Ciudad Real, Spain; 3 Centre for Ecology and Conservation, University of Exeter, Penryn, United Kingdom; 4 Centre d’Etudes Biologiques de Chizé, CNRS U.M.R. 7372 and Université de La Rochelle, Villiers-en-Bois, France; 5 Departamento de Ecología Evolutiva, Museo Nacional de Ciencias Naturales - CSIC, Madrid, Spain; University of Arkansas, UNITED STATES

## Abstract

Sexual selection promotes the evolution of conspicuous animal ornaments. To evolve as signals, these traits must reliably express the “quality” of the bearer, an indicator of individual fitness. Direct estimates of individual fitness may include the contribution of longevity and fecundity. However, evidence of a correlation between the level of signal expression and these two fitness components are scarce, at least among vertebrates. Relative fitness is difficult to assess in the wild as age at death and extra-pair paternity rates are often unknown. Here, in captive male red-legged partridges, we show that carotenoid-based ornament expression, i.e., redness of the bill and eye rings, at the beginning of reproductive life predicts both longevity (1–7 years) and lifetime breeding output (offspring number and hatching success). The recently proposed link between the individual capacity to produce red (keto) carotenoid pigments and the efficiency of cell respiration could, ultimately, explain the correlation with lifespan and, indirectly, fecundity. Nonetheless, in males of avian species, carotenoid-based coloration in bare parts is also partially controlled by testosterone. We also manipulated androgen levels throughout life by treating males with testosterone or antiandrogen compounds. Treatments caused correlations between signal levels and both fitness components to disappear, thus making the signals unreliable. This suggests that the evolution of carotenoid-based sexual signals requires a tightly-controlled steroid metabolism.

## Introduction

The ultimate and proximate mechanisms involved in the evolution of animal signals have been the subject of intense debate for decades (see e.g. recently [1–3]). Theoretical evolutionary models predict that signals must be reliable to evolve (e.g. [4, 5]). These reliable signals evolve when the signal production or maintenance costs are disproportionally high for low-quality individuals [6–8], or alternatively, when a direct causal link between signaling level and individual quality exists, making signals uncheatable [4, 9]. In both cases, those individuals producing larger or more intense signals should be higher-quality individuals, and be able to survive better and/or reproduce at a higher rate. In other words, higher signal expression should be linked to superior fitness, and not only as a result of direct benefits (fitness returns) of signaling (i.e. leading to a circular reasoning; [10]), but also to intrinsic genetic quality [4, 5, 11, 12]. The ornaments would thus became “honest” signals of high fitness [11–13].

Recently, the oxidative stress concept has gained empirical support, and is considered a ubiquitous selective pressure able to shape individual phenotypes from conception to death [14, 15]. Thus, it has been hypothesized that oxidative stress could be an important cost of reproduction influencing fecundity vs. survival trade-off [16–19]. That cost could not only be the consequence of increasing cell metabolism during reproduction (but see [19]) but also due to resource investments in sexual signals (e.g. [20]) that would then favor mating and reproductive success [15]. Among potential signals involved in the oxidative cost of reproduction, colored ornaments generated by carotenoids (many yellow-to-red traits) are probably the best known in vertebrates (e.g. [20–22]).

Carotenoid pigments are usually considered as antioxidants that can also be bleached by free radicals [22–24] (but see [25–27]). Accordingly, the production of carotenoid-based signals would risk the individual’s antioxidant status when no other compensatory mechanisms are available [22]. This would make carotenoid-based ornaments reliable signals of individual quality [22, 28]. Nevertheless, the positive links between the level of signal expression and longevity or fecundity has not definitively been established, but mostly inferred from other indicators of health status (reviewed in [24, 29, 30]) or, in the case of longevity, deduced from overwinter survival rates in avian species [31–36]. Although survival estimates in the wild are valuable as obtained under natural conditions, they are also subject to imperfect detection [37].

In captive conditions, zebra finches (*Taeinopygia guttata*) have been studied to understand the relationship between bill redness (a carotenoid-based signal) and longevity [38, 39]. One study reported no correlation among males, but a negative link in females [38], while another showed a positive association in both sexes [39]. Unfortunately, more than 40% of birds remained alive at the end of that study, and thus, an important part of individual variability (the oldest birds) was excluded (see [24] for additional analyses and data). As far as we know, the only longitudinal study that examined the association between carotenoid-based coloration and total lifetime longevity was performed by Pike *et al*. [40] in captive male sticklebacks (*Gasterosteus aculeatus*). In a one-year-long study, males with the reddest ventral skin at early life lived longer than paler ones in a population study of 34 individuals.

To our knowledge, the association between carotenoid-based signaling and lifetime reproductive success remains to be tested directly. In males, the association can be expected if we consider that a positive correlation between carotenoid-based color intensity and sperm quality has been detected [41, 42]. We must note that carotenoids are also present in semen [43], apparently protecting spermatozoids and germline DNA from oxidation [44].

In this framework, we consider the role of sex steroids. In males, testosterone is able to control/intensify carotenoid-based signal expression by increasing circulating carotenoid levels [45–48]. Sustaining high testosterone levels to favor carotenoid-based signaling theoretically implies costs derived from steroid pleiotropic effects such as injuries due to increased aggressiveness, immunosuppression or oxidative stress [49–51]. Alternatively, the level of expression of a sexual signal may predict the individual capacity to synthesize testosterone. McGlothlin *et al*. [52] found that male dark-eyed juncos (*Junco hyemalis*) with larger white plumage patches were more able to increase testosterone synthesis at a higher rate when triggered by gonadotropin injections. They argued that this capacity reinforced the link between signal size and fitness because testosterone improves reproductive success via increased mating effort, thus leading to a genetic correlation between signal size and testosterone levels (see also [53, 54]). However, testosterone levels may be subject to environmental influences that diminish this relationship. High population density and/or interaction rates among individuals can lead to sustained high testosterone levels that have been well demonstrated in mammals (see review in [55]) and birds [56–60]. Contrarily, breeding in dense populations can induce low testosterone levels in reptiles [55]. These studies illustrate the potential influence of environmental variability on testosterone levels, and theory suggests that environmental factors can alter the reliability of sexual signals, promoting or preventing their evolution (see [58, 61, 62]).

Here, we test whether male red carotenoid-based ornaments act as signals of fitness in the red-legged partridge, *Alectoris rufa*. The red-legged partridge is considered a monogamous species with long-term pair-bonds where intraspecific nest parasitism or polygamy can also occur [63, 64]. We previously found that wild birds mate assortatively according to trait redness [65], and captive females that mated with males whose carotenoid-based ornaments (red eye rings and bill) were artificially intensified laid more eggs [66]. These results suggest that these traits evolved as sexual signals involved in mate choice, and influenced post-mating resource allocation decisions in females. Here, we estimated the correlation between the expression level of these red traits at the first breeding age (one-year-old approx.) and total lifetime reproductive output over seven years. We must note that reproductive decisions at the first breeding opportunity may exert a crucial influence on life-history trajectories and fitness [67, 68]. Captive conditions allowed us to reliably establish both lifespan and paternity, as each male was individually housed with a female every reproductive season. Some of these males were treated throughout reproductive life with testosterone, vs those with anti-androgen compounds. The treatments allowed us to test the role of individual variability in testosterone levels in the link between signal level and lifetime fitness, mimicking the influence of environmental factors. We predicted that the intensity of the carotenoid-based coloration in controls should be positively correlated with longevity and fecundity, thus revealing individual quality [11–13]. We also predicted that lifetime testosterone action should disrupt that relationship. Based on previous evidence from this and other galliforms displaying carotenoid-based traits (e.g. [46, 58, 69, 70]), we predicted that if sustaining high testosterone levels implies higher costs for poor quality (paler) males [6, 7, 71], this correlation should increase in testosterone-treated males and, consequently, decrease in those receiving anti-androgens.

## Materials and methods

### Experimental protocol

This work was carried out at the Dehesa Galiana experimental facility (IREC, Ciudad Real, Spain) on captive-born, one-year old (born in spring 2005) red-legged partridges kindly provided by a large governmental breeding facility dedicated to restocking of wild populations (Chinchilla de Montearagón, Albacete, Spain; 38°55'13.6"N 1°42'40.3"W). The size of the population in this facility (850 breeding pairs and 10000 hatchlings produced per year) allowed us to choose males and females for this study, avoiding siblings in the sample. The same birds and dataset analyzed in Alonso-Alvarez et *al*. [72] were used here, but we assessed longevity and reproductive variables that were studied afterwards ending in 2013. Commercial pelleted food and water were provided ad libitum. Randomly formed pairs (*N* = 117) were kept in outdoor cages (1 × 0.5 × 0.4 m, length, width, height) under natural photoperiod and temperature conditions. Birds were acclimated from outdoor aviaries to cages for a minimum of 15 days before starting the experiment each year in April.

On April 10, 2006, a blood sample, body mass, and a color measurement (below) of each ornament, bill and eye ring, from each male partridge were taken. These were the initial values of this lifetime study. Blood samples were taken from the jugular vein within 2 min of the removal of a bird from its cage and stored at 4° C until centrifugation at 10,000 *g* for 10 min at 4°C to separate plasma from the cellular fraction. On April 20, 2006, males were subcutaneously implanted in the back 10 days after blood sampling with two silicone tubes (40 mm length, 1.47 mm i.d., 1.96 mm o.d.; Silastic tubing, Dow Corning, Midland, MI, USA). The date coincided with the time when the highest blood carotenoid concentrations were expected and when red carotenoid-based ornaments are displayed at the highest level and before egg laying began [73]. Males were randomly divided into four groups that received one of the four treatments. C-males (*n* = 30) received two empty implants. T-males (*n* = 29) received one of the implants packed with testosterone (Steraloids Inc.; Newport, RI, USA) plus the other empty one. F-treated males (*n* = 28) received an implant packed with flutamide (Sigma-Aldrich, St. Louis, MO, USA) and another empty one. Flutamide is a testosterone receptor blocker (e.g [74]). Finally, FA-males (*n* = 30) received an implant packed with flutamide plus an implant packed with 1,4,6-androstatriene-3,17-dione (ATD; Steraloids Inc.). ATD is a blocker of testosterone enzymatic transformation to estrogens (e.g [75]). The aim of the two anti-androgen treatments was to inhibit testosterone effects on target tissues. The FA implants would prevent not only direct effects on receptors but those due to estrogens (e.g [76]), which may increase as a result of testosterone-to-estradiol conversion when androgen receptors are neutralized (e.g [75, 77]). The tubes were closed at both ends with silicon glue (1 mm in each side; Nusil Technology, Carpinteria, CA, USA). The males were again sampled for blood and weighed 25 d and 70 d after the implant to control the effectiveness of hormonal treatments (15 May and 30 June, respectively; [72]).

The dimensions of the silicone tube were chosen from previous work on the same species that included a pilot study with same-sized implants (40 mm length) [45]. Blood hormone levels (see Hormonal phenotypes below) were within ranges described for non-manipulated red-legged partridges [78]. Testosterone and estradiol were measured by radioimmunoassay at the Centre d’Etudes Biologiques (Chizé, France; [79]).

The birds were released in two large adjacent outdoor aviaries (35 × 6 × 3 m; length, width, height; randomly distributed, with all groups represented) when the egg-laying period ended (10 July). Birds were again placed in the cages in March of the next year, sampled on 10 April and implanted on 20 April as in the preceding year. The same hormonal treatment and schedule was used for each bird during its entire life span. Implants were removed before being released to the aviaries. During the reproductive period, males were coupled with the same partner every year unless the female died or was injured and hence removed from the study. In these situations, the female was replaced by a yearling female without previous breeding experience obtained from the original population, i.e. Chinchilla de Montearagón (see above). The number of different females that mated with each male did not differ among treatments (Kruskal-Wallis test: *χ*^2^ = 3.13, df = 3, *P* = 0.372; range 1–6), or when that number was divided by the number of breeding events throughout the male’s life (*χ*^2^ = 0.57, df = 3, *P* = 0.904; see additional information in the supplementary material). The latter variable was also unrelated to lifetime hatching success of males (Spearman’s *r* = 0.079, *P* = 0.459).

Survivorship was checked twice weekly throughout the study. Egg laying was recorded, and eggs were moved from the cages to incubators daily (supplementary material). The number of chicks per cage was determined at the hatching date. “Lifetime hatching success” was the sum of all the hatchlings sired by a male divided by the sum of all the eggs produced by their mates. This variable was not calculated for males whose female(s) did not lay any egg throughout the experiment (supplementary material). We also determined “total chick survival” as the total number of chicks that reached 14d of life divided by the total number of hatchlings sired by the same male. Egg mass, hatchling mass and tarsus length, and chick body mass and tarsus length at 14d old were also measured.

### Color assessment

All individuals were photographed following Alonso-Alvarez and Galván [80] to analyze their ornamentation (see also [81]). We took digital photographs of red masks of birds with a digital camera (Olympus E-510) under standard bird position and light conditions. For each photo, the same standard gray reference scale (Kodak Gray Scale, Kodak, New York) was placed next to the bird’s neck. The color intensity of the bill and eye-rings of red-legged partridges was measured in photographs using Adobe Photoshop 7.0. For each individual, we calculated the RGB (red, green, blue) components of the eye ring and bill separately. Mean RGB values obtained for each trait per duplicate were repeatable (r > 0.82, *P* < 0.001; see [82]) and therefore, average values were used (see also [83] for repeatabilities and correlations between this and spectrophotometry-based methods). Hue values were determined by applying the Foley & van Dam algorithm [84] on RGB data. The covariation between trait hue values and those obtained from the reference chip was never significant (all *P* > 0.10). High values of hue indicated paler traits. For this reason, we reversed these hue values (multiplying by -1) to obtain a more intuitive variable, i.e. “redness”. To obtain positive values the variable was rescaled by adding 25, the minimum negative value. Thus, high eye ring or bill redness values indicate redder traits. Despite their similar carotenoid composition [85], eye ring and beak redness were treated separately because they differ in their responsiveness to variations in physiological status (e.g [86]). The photographs from two FA birds were lost when storing the digital file, and the bill of two control and one F bird could not be measured due to damage on the integument surface and were excluded from the analyses.

### Statistical analyses

Analyses were performed with IBM SPSS Statistics 24.0 (SPSS, Inc.). Cox proportional hazards models were applied to evaluate the influence of redness at the start of the reproductive life on longevity, and how hormone treatments may influence this potential association. Three birds (one C and two T birds) were excluded from the analyses as they escaped during the course of the experiment (also excluded in the dataset). In addition, another 14 individuals died due to accidents or their date of death could not be well established during the eight years of the study. The survivorship of these 14 birds until their last record was considered as censored data in log-rank tests and Cox models. The sample sizes of censored vs non-censored individuals were not biased among treatments (*χ*^2^ = 3.22, df = 3, *P* = 0.360). Linear and quadratic terms of the bill and eye ring redness relationship with survival were tested. The quadratic term was, however, always removed as it never reached the significance threshold (all *P*-values > 0.11).

The total number of eggs, lifetime hatching success and chick survivorship at 14 days of age showed zero-inflated distributions. Accordingly, Spearman’s correlation coefficients were used to analyze the relationship between redness and these reproductive variables. Moreover, we estimated the correlations between ornament redness and egg mass, hatchling and chick mass and tarsus length, all analyzed as mean values from lifetime data. Pearson’s correlation coefficients were used as these variables were normally distributed. The correlations between trait redness and reproductive parameters did not include censored data as we aimed to infer causal relationships between longevity and reproductive output. In any event, the same results were found when censored birds were included (S1 Table). In addition, correlations *P*-values were controlled for multiple testing by using the sequential Bonferroni procedure proposed by Benjamini and Hochberg [87], which considers the false discovery rate (FDR). In this case, the significance threshold moved from 0.05 to 0.009, thus only rejecting two tests from significance. We must, nonetheless, consider that we conventionally used two-tailed tests, whereas *a priori* predictions (see Introduction) would have allowed dividing the original *P*-values by two. This would only have removed from significance the correlation between eye-ring redness and number of eggs among F-males, thus not affecting the main conclusions of these results.

## Ethics

This investigation was approved by the institutional animal welfare committee (University of Castilla-La Mancha’s Committee on Ethics and Animal Experimentation) in accordance with pertinent Spanish legislation under Protocol number: 1011.01. The implanting procedure was performed under veterinary supervision. We used a planned humane endpoint where any bird would be immediately euthanized by cervical dislocation when it rapidly lost more than 20% of body mass.

## Results

### Hormonal phenotypes

Testosterone-treated males (S1A Fig) showed significantly higher testosterone values compared to all the other groups 25 d after the implant (see [72] for full statistical results). However, values of C- and F-males did not differ significantly [80], which was expected if no negative feedback on testosterone synthesis is triggered by androgen receptor blockage (e.g. [88]). In contrast, FA-males showed significantly higher testosterone concentrations than F- and C-males (S1A Fig; both *P*-values < 0.001; also in [72]). Higher plasma testosterone values in FA-males have repeatedly been reported in avian studies where the two compounds have simultaneously been used [75, 89–91]. Testosterone is used as the substrate to estradiol synthesis, and it is possible that a temporary decrease in estradiol levels due to ATD blockage could have triggered the production of its precursor (i.e. testosterone). The involvement of estradiol levels in this feedback would explain why F-only-males did not show higher testosterone levels. An increase in testosterone synthesis in FA-males could also explain their higher estradiol circulating levels compared to controls (S1B Fig). Regardless, the effect on estradiol was short-lived, disappearing in the following sampling event (see legend in S1 Fig).

### Redness and longevity

We detected a positive correlation between the intensity of the red-carotenoid based coloration in both head ornaments and lifespan (Table 1; omnibus tests: *χ*^2^ = 19.67, df = 7, *P* = 0.006 and *χ*^2^ = 18.77, df = 7, *P* = 0.009 for eye rings and bill models, respectively). Moreover, we found a significant interaction between hormonal treatments and redness in the eye ring (Table 1, *P* = 0.047). Pairwise comparisons among treatments revealed that individuals with redder eye rings and bills survived better than paler individuals among control males, and the slope of that relationship was significantly different from that of T-males, but from F- or FA-birds (Table 1).

**Table 1:** Cox proportional-hazards regression for survival.

**Eye ring redness**	**B**	**SE**	**Wald**	**df**	***P***	**Hazard ratio Exp(B)**	**95% CI**	
							**Lower**	**Upper**
Treatment			5.682	3	0.128			
Eye ring redness	0.317	0.122	6.701	1	**0.010**	0.729	0.573	0.926
Eye ring redness*treatment			7.961	3	**0.047**			
Eye ring redness*treatment (F)	-0.061	0.156	0.154	1	0.695	1.063	0.783	1.444
Eye ring redness*treatment (FA)	-0.129	0.167	0.594	1	0.441	1.138	0.819	1.580
Eye ring redness*treatment (T)	-0.479	0.184	6.761	1	**0.009**	1.615	1.125	2.318
**Bill redness**	**B**	**SE**	**Wald**	**df**	***P***	**Hazard ratio Exp(B)**	**95% CI**	
							**Lower**	**Upper**
Treatment			2.985	3	0.394			
Bill redness	0.205	0.068	9.142	1	**0.002**	8.15	0.713	0.930
Bill redness*treatment			4.851	3	0.183			
Bill redness*treatment (F)	-0.065	0.090	0.523	1	0.470	1.067	0.895	1.273
Bill redness*treatment (FA)	-0.094	0.109	0.737	1	0.391	1.098	0.887	1.360
Bill redness*treatment (T)	-0.197	0.092	4.585	1	**0.032**	1.218	1.017	1.459

^*^The relationship between trait redness and survival within flutamide (F), flutamide plus ATD (FA) or testosterone (T) groups using the same relationship in the control group as a reference.

*P*-values below 0.05 are shown in bold.

Table 1 shows the tests when using the controls as the reference group for pairwise comparisons. When the T-group is used instead (S2 Table), the relationship between eye ring redness and longevity also differs between T- and F-males (Wald test: 6.04, df: 1, *P* = 0.014), and showed a trend toward significance between the T- and FA-males (Wald test: 3.76, df: 1, *P* = 0.053). In the case of the bill, the slope of T-males did not significantly vary from that of F- or FA-treated males (both *P* > 0.13; also S2 Table). All of these differences lead to slopes that are ordered from steeper to flatter in the following way: C, F, FA and T (see Table 1). Finally, when Cox regressions were calculated separately for each treatment, the slope was significantly different from zero in C- and F-males, but not in FA- and T-treated males (Table 2), and the slopes were ordered in the same way.

**Table 2:** Cox proportional-hazards regression for survival and ornament (above eye ring, below bill) redness analyzed separately for each treatment. Control (C), flutamide (F), flutamide plus ATD (FA) and testosterone (T).

**Eye ring redness**	**B**	**SE**	**Wald**	**df**	***P***	**Hazard ratio Exp(B)**	**95% CI**	
							**Lower**	**Upper**
Control	0.368	0.133	7.688	1	**0.006**	0.692	-.368	0.133
Flutamide (F)	0.250	0.107	5.471	1	**0.019**	0.779	-.250	0.107
Flutamide + ATD (FA)	0.121	0.114	1.115	1	0.291	0.886	-.121	0.114
Testosterone (T)	-0.194	0.140	1.915	1	0.166	1.214	.194	0.140
**Bill redness**	**B**	**SE**	**Wald**	**df**	***P***	**Hazard ratio Exp(B)**	**95% CI**	
							**Lower**	**Upper**
Control	0.223	0.073	9.266	1	**0.002**	0.800	0.693	0.924
Flutamide (F)	0.140	0.067	4.390	1	**0.036**	0.869	0.762	0.991
Flutamide + ATD (FA)	0.086	0.083	1.062	1	0.303	0.918	0.780	1.080
Testosterone (T)	0.026	0.063	0.178	1	0.673	0.974	0.861	1.102

Control (C), flutamide (F), flutamide plus ATD (FA) and testosterone (T).

**Fig 1:**
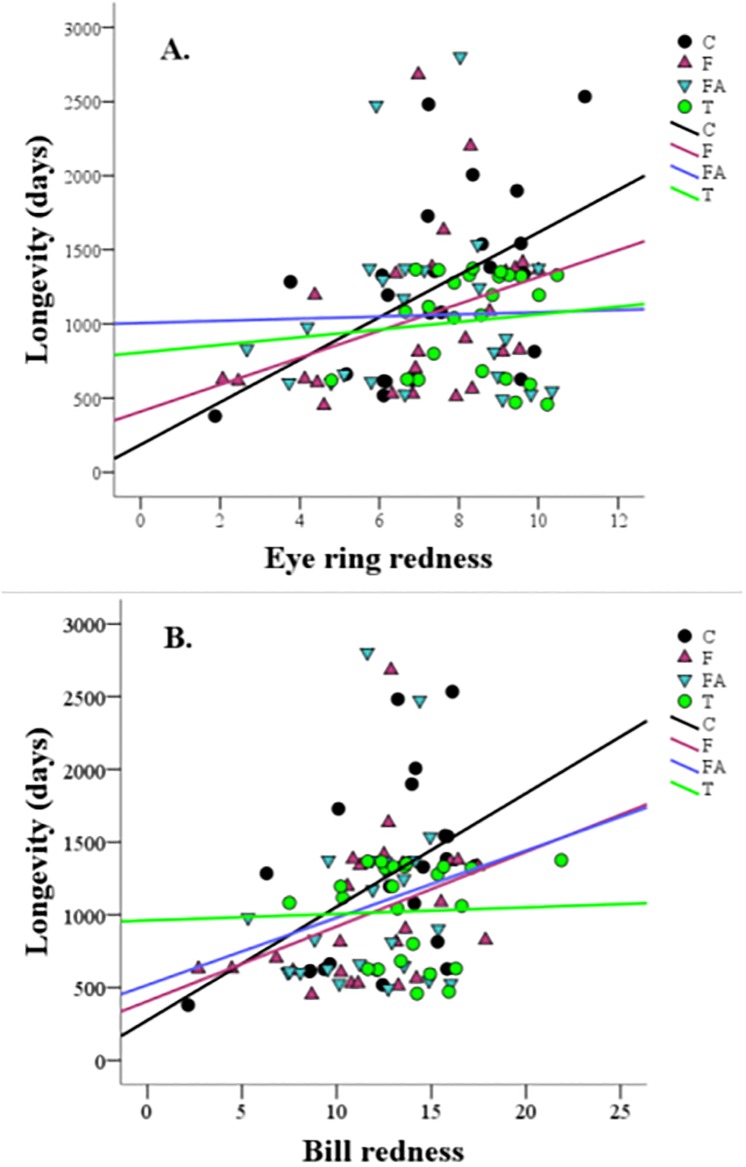
Relationship between longevity and eye ring (A) or bill (B) redness in male partridges with different hormonal treatments. Redness was measured before the first breeding opportunity. Slopes were obtained from ordinary least squares linear regressions. However, Cox regression slopes should be considered (see Results and Tables 1 and 2). Control (C), flutamide (F), flutamide plus ATD (FA) and testosterone (T) treatments. Censored data were excluded here.

Additionally, Fig 1 shows raw data and ordinary least squares linear regression slopes for illustrative purposes. The non-parametric Cox regression statistics were used to avoid the lack of normality and take into account censored data. The figure shows similar trends described by Cox regression statistics. Finally, survival plots dividing the sample by redness tertiles are also reported in the supplementary material (S2 and S3 Figs).

### Redness and lifetime reproductive success

**Fig 2:**
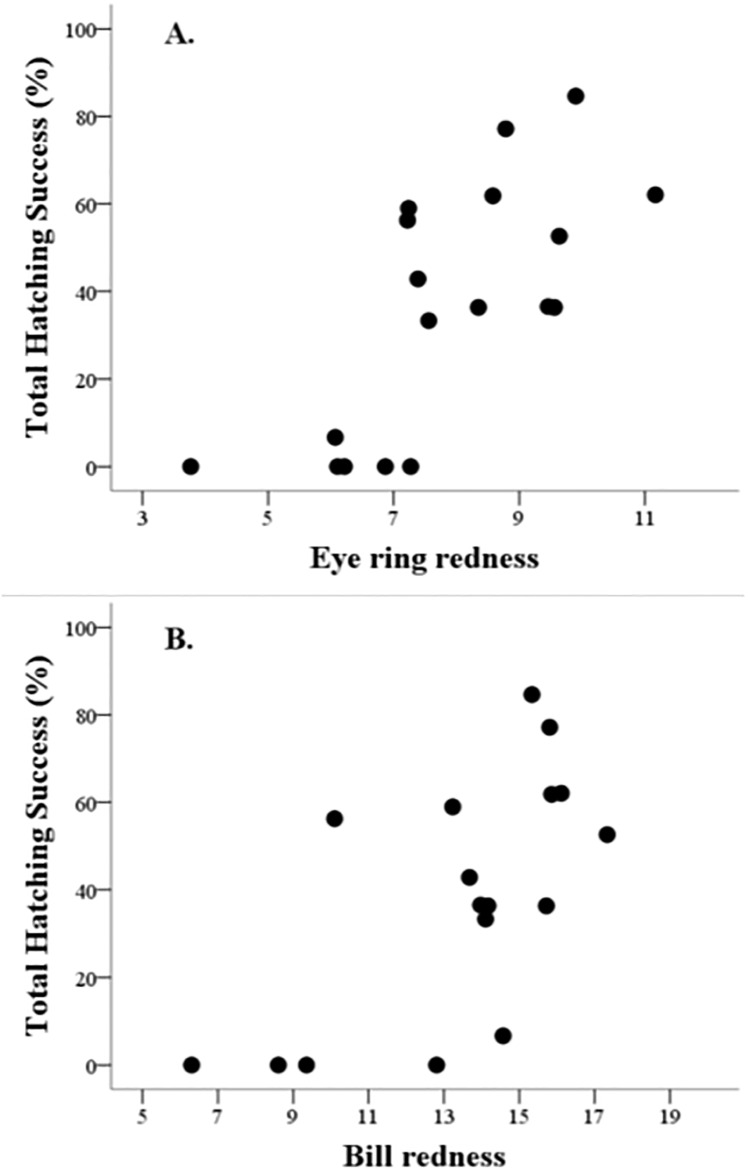
Relationship between total hatching success and eye ring (A) or bill (B) redness among control males. The total hatching success during lifetime was predicted by the redness of the eye ring (A) or bill (B) at the start of the first breeding season among control males.

Control birds showed significant positive correlations between bill and eye ring redness and the total number of eggs, hatchlings and 14 d-old chicks sired during life (Table 3). Interestingly, other groups did not show any of these relationships (Table 3), except F-males where the eye ring redness was also positively related to the total number of eggs and hatchlings (though the later at *P* = 0.071; Table 3). Moreover, lifetime hatching success also positively correlated with ornament redness, but again only among control males (Table 3 and Fig 2A and 2B). Finally, no significant correlation (all *P* > 0.10) was detected when between eye ring or bill redness and the mean value of egg, hatchling and chick mass and tarsus length during the lifetime in any group.

**Table 3:** Correlation between ornament redness and reproductive output in male red-legged partridges.

C-males		Number of eggs	Number of hatchlings	Number of 14d old chicks	Hatching success	Chick survivorship
Eye ring redness	*r*	0.538	0.638	0.555	0.717	-0.278
	*P*	**0.008**	**0.001**	**0.006**	**0.001**	0.357
	*n*	23	23	23	18	13
Bill redness	*r*	0.510	0.629	0.577	0.654	0.044
	*P*	**0.015**	**0.002**	**0.005**	**0.004**	0.886
	*n*	22	22	22	17	13
**F-males**						
Eye ring redness	*r*	0.417	0.360	0.249	-0.353	-0.363
	*P*	**0.034**	0.071	0.221	0.117	0.116
	*n*	26	26	26	21	20
Bill redness	*r*	0.216	0.311	0.258	0.073	-0.174
	*P*	0.301	0.131	0.213	0.760	0.477
	*n*	25	25	25	20	19
**FA-males**						
Eye ring redness	*r*	0.075	-0.040	-0.030	-0.301	0.111
	*P*	0.729	0.852	0.890	0.211	0.683
	*n*	24	24	24	19	16
Bill redness	*r*	0.211	0.088	0.063	-0.290	-0.068
	*P*	0.322	0.683	0.770	0.228	0.803
	*n*	24	24	24	19	16
**T-males**						
Eye ring redness	*r*	0.110	-0.069	-0.125	-0.006	-0.163
	*P*	0.602	0.742	0.550	0.979	0.632
	*n*	25	25	25	20	11
Bill redness	*r*	0.111	-0.023	-0.043	-0.095	0.060
	*P*	0.598	0.915	0.840	0.692	0.860
	*n*	25	25	25	20	11

Spearman’s correlation coefficients. *P*-values below 0.05 are shown in bold. *P*-values shown here are not controlled for multiple testing (see Statistical analyses).

## Discussion

We show that carotenoid-based colored ornaments can act as fitness signals because their intensity is positively correlated to both lifespan and lifetime fecundity. To our knowledge, this is the first study in any vertebrate species reporting a significant link between the expression level of a carotenoid-based colored ornament and longevity and lifetime fecundity. Simons et al. [39] reported these correlations but during a shorter period of life (part of the sample was still alive at the end of the study), and reproductive success in their study period, i.e. not lifetime, was estimated assuming that extrapair paternity did not affect their results (also [38]). However, they recognized that zebra finch extrapair paternity may reach 29% in similar aviary conditions (citing [92, 93], also Ana Angela Romero-Haro & Carlos Alonso-Alvarez, unpublished data). Here, we avoided this problem as only one pair was housed per cage, and the eggs were immediately removed and artificially incubated. Moreover, we show that the testosterone function throughout life may reduce the link between signal level and fitness, providing further support for a role of sex steroids in the evolution of carotenoid-based signals [45].

The mechanistic link between signal expression and longevity may rely on specific pathways leading to the production of carotenoid-based ornaments. The red traits of partridges are the result of the metabolic transformation of dietary yellow hydroxycarotenoids, mostly zeaxanthin and lutein, to red ketocarotenoids (i.e. astaxanthin and papilioerythrinone, respectively; [94]). It has been hypothesized that this type of transformation depends on oxidase (ketolase) enzymes [95, 96]. Johnson and Hill [97] proposed that ketolases could be located in the inner mitochondrial membrane, sharing catalytic reactions with cell respiration. This should establish a causal link between the level of signal expression and cell metabolism. Thus, rather than signals being sustained by their production costs, the red carotenoid-based ornaments would become indices of quality [9, 26, 27, 98, 99]. The well-known link between cell respiration and aging (e.g [100–102]) could, at least partially, explain the correlation between carotenoid-based signaling and longevity. A connection between red carotenoid-based coloration and cell respiration has been recently supported by a study in which male zebra finches exposed to a mitochondria-specific (targeted) antioxidant (mitoQ) produced redder bills [103]. Interestingly, the same antioxidant is able to extend lifespan in *Caenorhabditis elegans* [104].

In regard to fecundity, the correlation between redness and reproductive variables can arise as a consequence of longevity, as redder birds live longer and thus have more breeding opportunities. Accordingly, if the association between redness and egg or offspring numbers is controlled for longevity (i.e. by non-parametric partial correlations; see S3 and S4 Tables), all the tests became non-significant (all *P* > 0.18). This suggests that red ornaments signal fitness by informing about the individual capacity to live longer, which in turn allows higher fecundity. Thus, although an effect of male redness on female resource allocation to egg numbers has been reported in this species [66], this should not be the explanation for the present results. Instead, intrinsic genetic quality associated with longevity and probably supported by the mechanisms of ketocarotenoid production could be involved [9, 97, 103].

Less clear, however, is the mechanism underlying the link between redness and hatching success. Hatching success would control for the individual variability in the number of eggs and hence, longevity. Moreover, partial correlations controlling for longevity did not affect the statistical significance of the correlation (control birds, both ornaments: *P* < 0.017; see S3 Table). Here, candidate mechanisms may involve (1) variability in sperm quality perhaps related to carotenoid levels in semen (see e.g. [42]) and/or (2) maternal effects allowing females to produce eggs with higher quality when mated with redder males (see [66]). Nonetheless, our data do not allow to address any of these mechanisms.

Interestingly, the relationship between signal expression and fitness components disappeared when testosterone function was altered throughout the lifetime. The results followed the predictions in the case of anti-androgen treatments (particularly FA-birds) as the slope of the relationship became flatter. Contrarily, exogenous testosterone did not reinforce the link, i.e. promoting a steeper slope (see [69]). Paler males did not survive less than redder males when exposed to high testosterone levels, which should be expected if the hormone imposes disproportionally higher costs to the former [7, 51].

The lack of a reinforcing positive effect of the testosterone treatment on the correlation between signal expression and fitness may, nonetheless, be due to the effect of the exogenous hormone on fitness parameters. Testosterone implants reduced lifespan in this sample of birds (Carlos Alonso-Alvarez et al., unpublished results), which is consistent with the existence of physiological costs linked to sustaining high testosterone levels, such as immunosuppression [51, 105] (but see [106]) or oxidative stress [50, 69] (but see also [107]). This is consistent with reduced survival in wild birds treated with testosterone implants [49, 108–110]. However, male partridges implanted with testosterone also showed lower hatching success (Alonso-Alvarez et al., unpublished results) probably due to feedback inhibiting endogenous testosterone synthesis. This feedback was known to induce testes involution in different avian species [111–113] (but see [114]). A shorter variability in both lifespan and hatching success among T-males may have prevented statistically detection of a correlation between signal level and fitness in these birds, which would preclude definitive conclusions from this group of birds.

In any event, whatever the proximate mechanisms involved, the results show that variation in testosterone levels or activity during a lifetime can interfere with the reliability of signals. This implies that testosterone function should be tightly regulated to allow the evolution of red ketocarotenoid based signals. This suggests that the expression of these traits and testosterone/androgen receptor levels should be genetically correlated (see [53]). The demonstration of testosterone-mediated interference with carotenoid-based fitness signaling, while recognizing that it was performed under experimental hormone conditions, supports the hypothesized interconnection between pigment and sexual steroid-based mechanisms in the expression of sexual signals (see [45, 47, 115]). We should also note that selective pressures under free-living conditions would strongly differ from those in captivity. Thus, attaining extra-pair offspring would alter the result of the cost/benefit equations.

To summarize, our study reports a positive correlation between the level of expression of carotenoid-based ornaments and both the whole lifespan and lifetime fecundity probably for the first time in any vertebrate species (but see [40], for a correlation with full longevity in fishes). The interfering role of testosterone in these associations highlights the complexity of carotenoid-based signaling mechanisms and the involvement of different physiological pathways to sustain signal reliability. Further work is needed to determine if the carotenoid-based signal expression or the expression of those genes involved in the mechanism of signal production (e.g [95, 96, 116]) are genetically correlated to the capacity to synthesize testosterone or regulate its function (e.g. via androgen receptor expression/mutations, etc.).

## Supporting information

S1 Fig Testosterone (ng/ml; A) and estradiol (pg/ml; B) levels of male partridges 25 days after being subcutaneously treated with empty implants (control: C) or instead with implants filled with flutamide (F), flutamide + ATD (FA) or testosterone (T). Means ± SEs are shown for each treatment. Testosterone levels significantly differed among groups (see also Alonso-Alvarez et al. 2009), whereas only the difference between FA-males and controls in estradiol levels reached significance (*P* = 0.025, other comparisons *P* > 0.19). Estradiol levels at the end of the breeding season (70 days after the implant date) did not differ among groups (all contrasts: *P-*values > 0.27).
(DOC)

S2 Fig Survival trajectories of male red-legged partridges depending on the red intensity of their eye rings as divided by tertiles and experimental treatments. When testing differences among tertiles within each treatment group, significant rank tests (*P* < 0.05) were found for controls and F-treated males, and *P* = 0.068 in the case of FA-males.
(DOC)

S3 Fig Survival trajectories of male red-legged partridges depending on the red intensity of the bill as divided by tertiles and experimental treatments. When testing differences among tertiles within each treatment group, significant rank tests (*P* < 0.05) were found for controls and F-treated males only.
(DOC)

S1 Table Correlation between ornament redness and reproductive output in male red-legged partridges. Censored data (individuals dying from non-natural causes; see Methods) are included in this sample. Spearman’s correlation coefficients. *P*-values below 0.05 are shown in bold.
(DOC)

S2 Table Cox proportional-hazard regression for survival. The relationship between trait redness and survival within control (C), flutamide (F) or flutamide plus ATD (FA) groups is compared to the same relationship when tested in the testosterone (T) group. *P*-values below 0.05 are shown in bold.
(DOC)

S3 Table Partial correlation between ornament redness and reproductive output when controlling for longevity. Censored individuals were excluded. *P*-values below 0.05 are shown in bold. Degrees of freedom are reported.
(DOC)

S4 Table Partial correlation between trait redness and reproductive output when controlling for longevity, including censored data. Censored data (individuals dying from non-natural causes; see Methods) are here included in this sample. *P*-values below 0.05 are shown in bold. Degrees of freedom are reported.
(DOC)

S5 Table The number of different females engaged in reproduction with each individual male. (DOC)

S6 Table Number of different females engaged in reproduction with each individual male divided by the number of breeding events of each male. (DOC)

S1 Methods (DOCX)
