# Supplementary material for: Carotenoid-based coloration predicts both longevity and lifetime fecundity in male birds, but testosterone disrupts signal reliability
Source: PLoS One. 2019 Aug 23;14(8):e0221436. doi: 10.1371/journal.pone.0221436 (PMC6707625; doi:10.1371/journal.pone.0221436)
Supplement: S1 Fig — Testosterone levels significantly differed among groups (see also Alonso-Alvarez et al. 2009), whereas only the difference between FA-males and controls in estradiol levels reached significance (P = 0.025, other comparisons P > 0.19). Estradiol levels at the end of the breeding season (70 days after the implant date) did not differ among groups (all contrasts: P-values > 0.27). (DOC) [file pone.0221436.s001.doc]

**S1 Fig. Testosterone (ng/ml; A) and estradiol (pg/ml; B) levels of male partridges 25 days after being subcutaneously treated with empty implants (control: C) or instead with implants filled with flutamide (F), flutamide + ATD (FA) or testosterone (T). Means ± SEs are shown for each treatment.**

Testosterone levels significantly differed among groups (see also Alonso-Alvarez et al. 2009), whereas only the difference between FA-males and controls in estradiol levels reached significance (*P* = 0.025, other comparisons *P* > 0.19). Estradiol levels at the end of the breeding season (70 days after the implant date) did not differ among groups (all contrasts: *P-*values > 0.27).
