# Supplementary material for: Carotenoid-based coloration predicts both longevity and lifetime fecundity in male birds, but testosterone disrupts signal reliability
Source: PLoS One. 2019 Aug 23;14(8):e0221436. doi: 10.1371/journal.pone.0221436 (PMC6707625; doi:10.1371/journal.pone.0221436)
Supplement: S2 Fig — When testing differences among tertiles within each treatment group, significant rank tests (P < 0.05) were found for controls and F-treated males, and P = 0.068 in the case of FA-males. (DOC) [file pone.0221436.s002.doc]

**S2 Fig. Survival trajectories of male red-legged partridges depending on the red intensity of their eye rings as divided by tertiles and experimental treatments.**

When testing differences among tertiles within each treatment group, significant rank tests (*P* < 0.05) were found for controls and F-treated males, and *P* = 0.068 in the case of FA-males.
