# Supplementary material for: Carotenoid-based coloration predicts both longevity and lifetime fecundity in male birds, but testosterone disrupts signal reliability
Source: PLoS One. 2019 Aug 23;14(8):e0221436. doi: 10.1371/journal.pone.0221436 (PMC6707625; doi:10.1371/journal.pone.0221436)
Supplement: S1 Table — Spearman’s correlation coefficients. P-values below 0.05 are shown in bold. (DOC) [file pone.0221436.s004.doc]

**S1 Table. Correlation between ornament redness and reproductive output in male red-legged partridges. Censored data (individuals dying from non-natural causes; see Methods) are included in this sample.**

Spearman’s correlation coefficients. *P*-values below 0.05 are shown in bold.

| **C-males** |  | **Number of eggs** | | **Number of hatchlings** | **Number of 14d old chicks** | **Hatching success** | **Chick survivorship** |
| --- | --- | --- | --- | --- | --- | --- | --- |
| Eye ring redness | *r* | 0.339 | | 0.407 | 0.345 | 0.465 | -0.308 |
|  | *P* | 0.072 | | **0.028** | 0.067 | **0.034** | 0.265 |
|  | *n* | 29 | | 29 | 29 | 21 | 15 |
| Bill redness | *r* | 0.512 | | 0.644 | 0.596 | 0.7 | -0.115 |
|  | *P* | **0.006** | | **0.000** | **0.001** | **0.001** | 0.697 |
|  | *n* | 27 | | 27 | 27 | 19 | 14 |
| **F-males** |  |  | |  |  |  |  |
| Eye ring redness | *r* | 0.371 | | 0.324 | 0.239 | -0.329 | -0.299 |
|  | *P* | 0.052 | | 0.093 | 0.222 | 0.135 | 0.188 |
|  | *n* | 28 | | 28 | 28 | 22 | 21 |
| Bill redness | *r* | 0.135 | | 0.244 | 0.211 | 0.097 | -0.084 |
|  | *P* | 0.501 | | 0.220 | 0.291 | 0.676 | 0.724 |
|  | *n* | 27 | | 27 | 27 | 21 | 20 |
| **FA-males** |  |  | |  |  |  |  |
| Eye ring redness | *r* | 0.110 | | -0.009 | -0.009 | -0.295 | 0.085 |
|  | *P* | 0.577 | | 0.964 | 0.965 | 0.171 | 0.728 |
|  | *n* | 28 | | 28 | 28 | 23 | 19 |
| Bill redness | *r* | 0.211 | | 0.142 | 0.082 | -0.123 | -0.128 |
|  | *P* | 0.280 | | 0.472 | 0.677 | 0.576 | 0.603 |
|  | *n* | 28 | | 28 | 28 | 23 | 19 |
| **T-males** |  |  | |  |  |  |  |
| Eye ring redness | *r* | 0.143 | | 0.004 | -0.052 | 0.069 | -0.163 |
|  | *P* | 0.476 | | 0.982 | 0.797 | 0.765 | 0.632 |
|  | *n* | 27 | | 27 | 27 | 21 | 11 |
| Bill redness | *r* | | 0.111 | 0.022 | -0.004 | -0.038 | 0.060 |
|  | *P* | | 0.582 | 0.915 | 0.985 | 0.868 | 0.860 |
|  | *n* | | 27 | 27 | 27 | 21 | 11 |
