# Supplementary material for: Carotenoid-based coloration predicts both longevity and lifetime fecundity in male birds, but testosterone disrupts signal reliability
Source: PLoS One. 2019 Aug 23;14(8):e0221436. doi: 10.1371/journal.pone.0221436 (PMC6707625; doi:10.1371/journal.pone.0221436)
Supplement: S2 Table — The relationship between trait redness and survival within control (C), flutamide (F) or flutamide plus ATD (FA) groups is compared to the same relationship when tested in the testosterone (T) group. P-values below 0.05 are shown in bold. (DOC) [file pone.0221436.s005.doc]

**S2 Table. Cox proportional-hazard regression for survival.**

| | **Eye ring redness** | | **B** | **SE** | **Wald** | **df** | ***P*** | **Hazard ratio Exp(B)** | **95% CI** | | | --- | --- | --- | --- | --- | --- | --- | --- | --- | --- | | **Lower** | **Upper** | | Treatment | |  |  | 5.682 | 3 | 0.128 |  |  |  | | Eye ring redness | | 0.163 | 0.136 | 1.442 | 1 | 0.230 | 1.177 | 0.902 | 1.535 | | **Eye ring redness*treatment** | |  |  | 7.961 | 3 | **0.047** |  |  |  | |  | **Eye ring redness*treatment (C)** | -0.479 | 0.184 | 6.761 | 1 | **0.009** | 0.619 | 0.431 | 0.889 | |  | **Eye ring redness*treatment (F)** | -0.418 | 0.170 | 6.044 | 1 | **0.014** | 0.658 | 0.472 | 0.919 | |  | Eye ring redness*treatment(FA) | -0.350 | 0.181 | 3.759 | 1 | 0.053 | 0.705 | 0.494 | 1.004 | | **Bill redness** | |  |  |  |  |  | **Hazard ratio Exp(B)** | **95% CI** | | | **B** | **SE** | **Wald** | **df** | ***P*** | **Lower** | **Upper** | | Treatment | |  |  | 2.985 | 3 | 0.394 |  |  |  | | Bill redness | | -0.008 | 0.063 | 0.015 | 1 | 0.901 | 0.992 | 0.877 | 1.122 | | Bill redness*treatment | |  |  | 4.851 | 3 | 0.183 |  |  |  | |  | Bill redness*treatment (C) | -0.197 | 0.092 | 4.585 | 1 | **0.032** | 0.821 | 0.685 | 0.983 | |  | Bill redness*treatment (F) | -0.132 | 0.087 | 2.285 | 1 | 0.131 | 0.876 | 0.738 | 1.040 | |
| --- | --- | --- | --- | --- | --- | --- | --- | --- | --- | --- | --- | --- | --- | --- | --- | --- | --- | --- | --- | --- | --- | --- | --- | --- | --- | --- | --- | --- | --- | --- | --- | --- | --- | --- | --- | --- | --- | --- | --- | --- | --- | --- | --- | --- | --- | --- | --- | --- | --- | --- | --- | --- | --- | --- | --- | --- | --- | --- | --- | --- | --- | --- | --- | --- | --- | --- | --- | --- | --- | --- | --- | --- | --- | --- | --- | --- | --- | --- | --- | --- | --- | --- | --- | --- | --- | --- | --- | --- | --- | --- | --- | --- | --- | --- | --- | --- | --- | --- | --- | --- | --- | --- | --- | --- | --- | --- | --- | --- | --- | --- | --- | --- | --- | --- | --- | --- | --- | --- | --- | --- | --- | --- | --- | --- | --- | --- | --- | --- | --- | --- | --- | --- | --- | --- | --- | --- | --- | --- | --- |

The relationship between trait redness and survival within control (C), flutamide (F) or flutamide plus ATD (FA) groups is compared to the same relationship when tested in the testosterone (T) group. *P*-values below 0.05 are shown in bold.
