# Supplementary material for: Carotenoid-based coloration predicts both longevity and lifetime fecundity in male birds, but testosterone disrupts signal reliability
Source: PLoS One. 2019 Aug 23;14(8):e0221436. doi: 10.1371/journal.pone.0221436 (PMC6707625; doi:10.1371/journal.pone.0221436)
Supplement: S3 Table — P-values below 0.05 are shown in bold. Degrees of freedom are reported. (DOC) [file pone.0221436.s006.doc]

***S3 Table. Partial correlation between ornament redness and reproductive output when controlling for longevity. Censored individuals were excluded.***

| **C-males** |  | **Number of eggs** | **Number of hatchlings** | **Number of 14d old chicks** | **Hatching success** | **Chick survivorship** |
| --- | --- | --- | --- | --- | --- | --- |
| Eye ring redness | *r* | -0.004 | 0.133 | 0.195 | 0.662 | -0.388 |
|  | *P* | 0.987 | 0.557 | 0.385 | **0.004** | 0.213 |
|  | *d.f.* | 20 | 20 | 20 | 15 | 10 |
| Bill redness | *r* | -0.091 | 0.036 | 0.123 | 0.590 | -0.183 |
|  | *P* | 0.694 | 0.877 | 0.594 | **0.016** | 0.570 |
|  | *d.f.* | 19 | 19 | 19 | 14 | 10 |
| **F-males** |  |  |  |  |  |  |
| Eye ring redness | *r* | 0.239 | 0.031 | 0.113 | -0.480 | -0.222 |
|  | *P* | 0.251 | 0.884 | 0.591 | **0.032** | 0.361 |
|  | *d.f.* | 23 | 23 | 23 | 18 | 17 |
| Bill redness | *r* | -0.017 | 0.037 | 0.138 | -0.071 | -0.032 |
|  | *P* | 0.937 | 0.862 | 0.521 | 0.772 | 0.899 |
|  | *d.f.* | 22 | 22 | 22 | 17 | 16 |
| **FA-males** |  |  |  |  |  |  |
| Eye ring redness | *r* | 0.289 | 0.046 | 0.090 | -0.274 | 0.077 |
|  | *P* | 0.181 | 0.836 | 0.683 | 0.271 | 0.784 |
|  | *d.f.* | 21 | 21 | 21 | 16 | 13 |
| Bill redness | *r* | 0.194 | -0.006 | 0.040 | -0.162 | 0.008 |
|  | *P* | 0.375 | 0.977 | 0.858 | 0.521 | 0.979 |
|  | *d.f.* | 21 | 21 | 21 | 16 | 13 |
| **T-males** |  |  |  |  |  |  |
| Eye ring redness | *r* | 0.058 | -0.252 | -0.267 | 0.011 | -0.561 |
|  | *P* | 0.788 | 0.235 | 0.207 | 0.964 | 0.092 |
|  | *d.f.* | 22 | 22 | 22 | 17 | 8 |
| Bill redness | *r* | 0.153 | -0.298 | -0.271 | -0.213 | 0.102 |
|  | *P* | 0.474 | 0.157 | 0.199 | 0.380 | 0.779 |
|  | *d.f.* | 22 | 22 | 22 | 17 | 8 |

*P*-values below 0.05 are shown in bold. Degrees of freedom are reported.
