# Supplementary material for: Carotenoid-based coloration predicts both longevity and lifetime fecundity in male birds, but testosterone disrupts signal reliability
Source: PLoS One. 2019 Aug 23;14(8):e0221436. doi: 10.1371/journal.pone.0221436 (PMC6707625; doi:10.1371/journal.pone.0221436)
Supplement: S4 Table — P-values below 0.05 are shown in bold. Degrees of freedom are reported. (DOC) [file pone.0221436.s007.doc]

**S4 Table. Partial correlation between trait redness and reproductive output when controlling for longevity, including censored data. Censored data (individuals dying from non-natural causes; see Methods) are here included in this sample.**

| **C-males** |  | **Number of eggs** | **Number of hatchlings** | **Number of 14d old chicks** | **Hatching success** | **Chick survivorship** |
| --- | --- | --- | --- | --- | --- | --- |
| Eye ring redness | *r* | 0.059 | 0.134 | 0.171 | 0.358 | -0.309 |
|  | *P* | 0.764 | 0.496 | 0.385 | 0.121 | 0.283 |
|  | *d.f.* | 26 | 26 | 26 | 18 | 12 |
|  |  |  |  |  |  |  |
| Bill redness | *r* | -0.053 | 0.072 | 0.130 | 0.594 | -0.211 |
|  | *P* | 0.797 | 0.727 | 0.527 | **0.009** | 0.488 |
|  | *d.f.* | 24 | 24 | 24 | 16 | 11 |
| **F-males** |  |  |  |  |  |  |
| Eye ring redness | *r* | 0.207 | 0.031 | 0.117 | -0.438 | -0.153 |
|  | *P* | 0.300 | 0.879 | 0.560 | **0.047** | 0.520 |
|  | *d.f.* | 25 | 25 | 25 | 19 | 18 |
| Bill redness | *r* | -0.050 | 0.026 | 0.129 | -0.040 | 0.024 |
|  | *P* | 0.809 | 0.899 | 0.531 | 0.868 | 0.923 |
|  | *d.f.* | 24 | 24 | 24 | 18 | 17 |
| **FA-males** |  |  |  |  |  |  |
| Eye ring redness | *r* | 0.161 | -0.057 | 0.016 | -0.254 | 0.144 |
|  | *P* | 0.421 | 0.778 | 0.936 | 0.254 | 0.568 |
|  | *d.f.* | 25 | 25 | 25 | 20 | 16 |
| Bill redness | *r* | 0.196 | 0.075 | 0.065 | -0.064 | -0.097 |
|  | *P* | 0.327 | 0.709 | 0.747 | 0.776 | 0.703 |
|  | *d.f.* | 25 | 25 | 25 | 20 | 16 |
| **T-males** |  |  |  |  |  |  |
| Eye ring redness | *r* | 0.204 | -0.111 | -0.135 | 0.053 | -0.561 |
|  | *P* | 0.316 | 0.589 | 0.510 | 0.825 | 0.092 |
|  | *d.f.* | 24 | 24 | 24 | 18 | 8 |
| Bill redness | *r* | 0.193 | -0.261 | -0.239 | -0.198 | 0.102 |
|  | *P* | 0.345 | 0.197 | 0.239 | 0.402 | 0.779 |
|  | *d.f.* | 24 | 24 | 24 | 18 | 8 |

*P*-values below 0.05 are shown in bold. Degrees of freedom are reported.
