# Supplementary material for: Carotenoid-based coloration predicts both longevity and lifetime fecundity in male birds, but testosterone disrupts signal reliability
Source: PLoS One. 2019 Aug 23;14(8):e0221436. doi: 10.1371/journal.pone.0221436 (PMC6707625; doi:10.1371/journal.pone.0221436)
Supplement: S5 Table — (DOC) [file pone.0221436.s008.doc]

S5 Table. The number of different females engaged in reproduction with each individual male:

| **TREATMENT** | **Mean** | **RANGE** |
| --- | --- | --- |
| CONTROL | 2 | 5 |
| F-MALES | 1.6 | 2 |
| FA-MALES | 1.7 | 4 |
| T-MALES | 1.5 | 3 |
