# Supplementary material for: Carotenoid-based coloration predicts both longevity and lifetime fecundity in male birds, but testosterone disrupts signal reliability
Source: PLoS One. 2019 Aug 23;14(8):e0221436. doi: 10.1371/journal.pone.0221436 (PMC6707625; doi:10.1371/journal.pone.0221436)
Supplement: S6 Table — (DOC) [file pone.0221436.s009.doc]

S6 Table: Number of different females engaged in reproduction with each individual male divided by the number of breeding events of each male:

| **TREATMENT** | **Mean** | **RANGE** |
| --- | --- | --- |
| CONTROL | 0.83 | 1.67 |
| F-MALES | 0.79 | 0.67 |
| FA-MALES | 0.77 | 1.50 |
| T-MALES | 0.75 | 1 |
