# Supplementary material for: Carotenoid-based coloration predicts both longevity and lifetime fecundity in male birds, but testosterone disrupts signal reliability
Source: PLoS One. 2019 Aug 23;14(8):e0221436. doi: 10.1371/journal.pone.0221436 (PMC6707625; doi:10.1371/journal.pone.0221436)
Supplement: S1 Methods — (DOCX) [file pone.0221436.s010.docx]

**S1 Methods**

The birds in our study came from a very large captive population (Granja Cinegética de Chinchilla de Montearagón; Junta de Comunidades de Castilla-La Mancha, JCCM; Albacete, SE Spain; 38°55'13.6"N 1°42'40.3"W) of about 850 breeding pairs that approximately produces 10.000 hatchlings per year. The cited population reinforces its numbers by taking eggs (several hundred every year) from a large wild population (15000 hectares). The activity of the cited farm is devoted to restocking of wild populations for game activities in Spain (about 3 millions of birds are released every year from different commercial farms established across the Iberian Peninsula). The Castilla-La Mancha (Central Spain) government provided us with birds yearly, which were always randomly chosen for us. This allowed us to avoid including siblings in our study design.

The females used to replace casualties were always born the year before and had no previous breeding experience. They were always randomly taken from the replacement pool described above. Moreover, we have tested the number of different females engaged in reproduction with each individual male. There were no statistical differences among treatments (mentioned at the original version), even when controlled for the number of breeding events of each male (note that T-males lived less). This is now reported and emphasized in the main text (Lines 245-248). We have no a priori reasons to believe that bias could exist.

**Reproduction monitoring**

Eggs were daily identified with a pencil and then stored at 15ºC. At this temperature, embryo development is arrested [1]. Stored eggs were transferred to incubators (37ºC) every 15 d. Six incubation periods (24 d each) were programmed. Synchronizing hatching events was necessary to allow the management of large numbers of chicks in captive conditions [2]. The chicks were individually identified by means of Velcro © numbered rings in the tarsus, which were substituted to larger plastic rings at 14d old. The Velcro rings were fixed with staples and reviewed every two days.

The proportion of males that did not sire eggs (19.8%) was equally distributed among treatments (*χ*^2^ = 0.70, df = 3, *P* = 0.995).

1. Thear K. Incubation: a guide to hatching and rearing. Broad Leys Publishing, Essex.1987.

2. Alonso-Alvarez C, Pérez‐Rodríguez L, García JT, Viñuela J, Mateo R. Age and breeding effort as sources of individual variability in oxidative stress markers in a bird species. Physiological and Biochemical Zoology. 2010;83(1):110-8. doi: 10.1086/605395. PubMed PMID: 19922287.
